# Parental experiences of homeschooling during the COVID-19 pandemic: differences between seven European countries and between children with and without mental health conditions

**DOI:** 10.1007/s00787-020-01706-1

**Published:** 2021-01-07

**Authors:** Lisa B. Thorell, Charlotte Skoglund, Almudena Giménez de la Peña, Dieter Baeyens, Anselm B. M. Fuermaier, Madeleine J. Groom, Irene C. Mammarella, Saskia van der Oord, Barbara J. van den Hoofdakker, Marjolein Luman, Débora Marques de Miranda, Angela F. Y. Siu, Ricarda Steinmayr, Iman Idrees, Lorrayne Stephane Soares, Matilda Sörlin, Juan Luis Luque, Ughetta M. Moscardino, Maja Roch, Giulia Crisci, Hanna Christiansen

**Affiliations:** 1 Division of Psychology, Department of Clinical Neuroscience, Karolinska Institutet, Nobels Väg 9, 171 77 Stockholm, Sweden; 2 Department of Clinical Neuroscience, Karolinska Institutet, Stockholm, Sweden; 3 University of Málaga, Málaga, Spain; 4 KU Leuven, Leuven, Belgium; 5 University of Groningen, Groningen, The Netherlands; 6 University of Nottingham, Nottingham, UK; 7 University of Padua, Padua, Italy; 8 University Medical Center Groningen and University of Groningen, Groningen, The Netherlands; 9 Vrije Universiteit Amsterdam, Amsterdam, The Netherlands; 10 University Federal de Minas Gerais, Belo Horizonte, Brazil; 11 The Chinese University of Hong Kong, Hong Kong, China; 12 TU Dortmund, Dortmund, Germany; 13 Philipps University Marburg, Marburg, Germany

**Keywords:** Homeschooling, COVID-19, Mental health problems, Parental experiences, Special education needs

## Abstract

The aim of the present study was to examine parental experiences of homeschooling during the COVID-19 pandemic in families with or without a child with a mental health condition across Europe. The study included 6720 parents recruited through schools, patient organizations and social media platforms (2002 parents with a child with a mental health condition and 4718 without) from seven European countries: the UK (*n* = 508), Sweden (*n* = 1436), Spain (*n* = 1491), Belgium (*n* = 508), the Netherlands (*n* = 324), Germany (*n* = 1662) and Italy (*n* = 794). Many parents reported negative effects of homeschooling for themselves and their child, and many found homeschooling to be of poor quality, with insufficient support from schools. In most countries, contact with teachers was limited, leaving parents with primary responsibility for managing homeschooling. Parents also reported increased levels of stress, worry, social isolation, and domestic conflict. A small number of parents reported increased parental alcohol/drug use. Some differences were found between countries and some negative experiences were more common in families with a child with a mental health condition. However, differences between countries and between families with and without a mental health condition were generally small, indicating that many parents across countries reported negative experiences. Some parents also reported positive experiences of homeschooling. The adverse effects of homeschooling will likely have a long-term impact and contribute to increased inequalities. Given that school closures may be less effective than other interventions, policymakers need to carefully consider the negative consequences of homeschooling during additional waves of the COVID-19 pandemic and future pandemics.

## Introduction

The medical consequences of COVID-19 have been severe, but little is known about the consequences for the daily life functioning of families and children. In particular, to reduce COVID-19 transmission, many countries imposed lockdown measures, including school closures. Schools offer many critical services beyond education (e.g., nutrition, exercise, social contact, and mental health services) [1] and school closures may therefore disrupt the everyday functioning of children and their parents. A few recent reviews and commentaries have suggested that social isolation contributes to depression [2] and may contribute to mental health risks for children (e.g., stress, anxiety, family conflict) both during and after the pandemic [3]. There are also empirical data showing that during the COVID-19 pandemic, behavioral problems (e.g., irritability/aggression, inattention and internalizing problems) have been common in children [4, 5] and homeschooling is one of the activities associated with the strongest negative effects [5]. Nonetheless, there have been no large-scale studies including several different countries, which have examined the experiences of homeschooling during the COVID-19 outbreak.

It has been hypothesized that children with mental health conditions may be especially vulnerable to the consequences of the COVID-19 pandemic [6, 7]. A few smaller surveys targeting parents of children with neurodevelopmental disorders have shown increased problems with managing daily life, aggression, and home schooling [8–10]. However, it has also been suggested [3, 11] that children with mental health conditions may experience positive aspects of homeschooling—a claim requiring further exploration.

The overall aim of the present study was to investigate parents’ experiences of homeschooling and their perceptions of the effects of school closures on daily life for themselves and their child during the COVID-19 pandemic (i.e., April through June, 2020). As countries vary with regard to restriction levels and schooling organization during the COVID-19 pandemic, which may have different impacts on functioning, data from seven different European countries were included. To investigate families that may be particularly vulnerable to the consequences of the COVID-19 pandemic, we oversampled families with a child with mental health problems. First, we examined differences between seven European countries with regard to the following research questions: (1) how was homeschooling during the COVID-19 pandemic organized in terms of teaching in general (i.e., on-line teaching, parent-led homeschooling, peer-led homeschooling, self-study) and support for children with special educational needs? (2) What negative and positive experiences of homeschooling did parents report during the COVID-19 pandemic? (3) To what extent did parents experience changes in daily life functioning (e.g., family conflicts, parental alcohol use) when comparing their current situation with life before the pandemic? Second, we compared families with and without a child with mental health problems on parental experiences of homeschooling and changes in daily life functioning during the COVID-19 pandemic.

## Methods

### Participants

The inclusion criterion for the present study was parent of a child (age 5–19 years) receiving homeschooling due to school closure during the COVID-19 pandemic. If a parent had more than one child doing homeschooling, they were asked to rate their eldest child. Altogether, the study included 6720 parents from seven countries across Europe: the United Kingdom (UK; *n* = 508), Sweden (*n* = 1432), Spain (*n* = 1491), Belgium (*n* = 508), the Netherlands (*n* = 324), Germany (*n* = 1662), and Italy (*n* = 794). Parents were asked whether their child had received a mental health diagnosis. If answering “yes”, they were asked to list what diagnosis/diagnoses the child had received. To ensure these families were sufficiently well-represented in the dataset, we advertised the study via support groups and social media forums that cater specifically to families affected by mental health problems in general or neurodevelopmental disorders specifically [i.e. Attention Deficit Hyperactivity Disorder (ADHD) or Autism Spectrum Disorders (ASD)]. A total of 2002 parents of children diagnosed with at least one mental health condition (MHC group) and 4718 without a mental health condition (NO-MHC) were included in the study.

### Materials and procedure

**Fig. 1:**
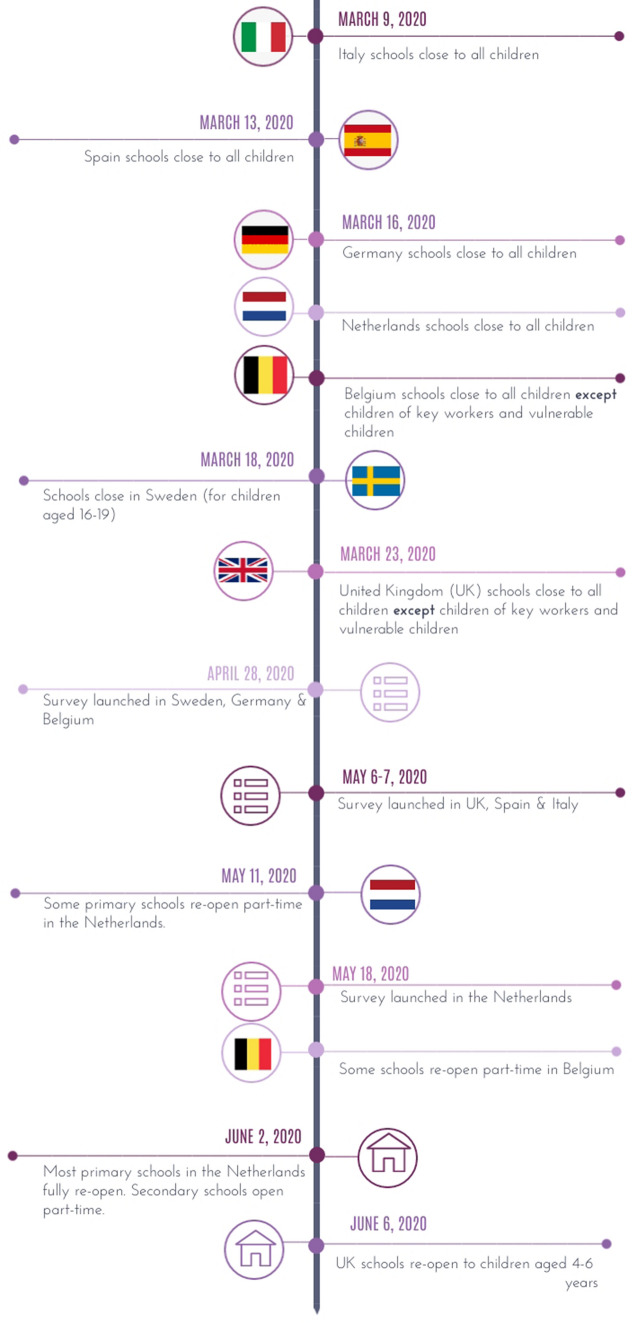
Timeline of school closures and survey data collection March to June 2020. The figure shows the timing of key events in relation to school closures and, where applicable, the reopening of schools in the 7 countries that took part in the survey. Unless otherwise stated, schools did not reopen fully before the summer holidays in each country

Data were collected from April 28 to June 21, 2020, using an anonymous digital survey distributed to parents via social media, schools, parent networks, and parent support groups (see Fig. 1 for a timeline of the study). A wide range of schools from diverse socio-economic areas in each country were asked to support the study by sharing the survey with parents. Families with mental health problems were oversampled in all countries, except Germany and Italy. This oversampling was achieved by posting information about the study on various social media forums targeting mental health problems in general or forums or support groups specifically targeting Attention Deficit Hyperactivity Disorder (ADHD) and/or autism spectrum disorders (ASD). The study was approved by the ethics committees in each one of the seven participating countries.

#### Organization of homeschooling

Organization of homeschooling was measured as the percentage of time spent on the following activities: (1) teacher contact (i.e., on-line teaching), (2) peer contact, (3) self-study and (4) parent contact. Secondly, we asked parents whether their child normally received special educational support and to what extent extra support was received during homeschooling. For parents whose child did receive extra support during homeschooling, we also asked whether they thought that this support was sufficient and whether the school had been in contact with the family to discuss the need for support during homeschooling.

#### Negative and positive experiences of homeschooling

Negative and positive experiences of homeschooling focused on the following four domains: (1) quality of homeschooling, (2) general negative and positive experiences of homeschooling, (3) parental worry/stress and (4) child participation in homeschooling. We created the survey based on themes that had been identified as being of most importance in a smaller qualitative survey where parents were asked to more freely describe negative and positive experiences of homeschooling during the COVID-19 pandemic. Quality of homeschooling was measured using the following 3 items: “the quality of my child’s homeschooling is very poor”, “The school’s support to students during homeschooling is not sufficient” and “It is impossible to get homeschooling to work well for my child.” General experiences included two items each for children (“Homeschooling has had negative/positive effects on my child’s life”) and parents (“Homeschooling has had positive/negative effects on my own life”). The item “I am worried that my child will not be able to handle school as well as he/she normally does because of homeschooling” measured parental worry and the item” I feel stressed because of the extra work that homeschooling demands of me as a parent” measured parental stress. Child participation in homeschooling was measured using the following two items:” Homeschooling puts too high demands on my child to plan his/her own schoolwork” and” My child cannot fully take part in homeschooling and therefore misses some of the school activities.” A 5-point Likert scale ranging from 1 (“strongly disagree) to 5 (“strongly agree”) was used for all items. However, when analyzing the data, all items were dichotomized and reported as the proportion of parents reporting the two highest scores (i.e., “agree” or “strongly agree”) as this provided a simple metric to compare between countries and between MHC and NO-MHC families.

#### Changes on daily life functioning during the COVID-19 pandemic

With regard to negative changes on daily life functioning, we included a number of questions assessing changes during the COVID-19 pandemic compared to before using a 5-point scale (1 = “much less than before” and 5 = “much more than before”). These questions assessed social isolation, family conflict, alcohol use, child digital media use, parental work problems and financial problems. The specific items included to measure each domain are presented in Table 4. Similarly to the items measuring parental experiences of homeschooling, all items were dichotomized and analyzed as the proportion of parents reporting the two highest scores.

### Statistical analyses

First, we used Analyses of Variance (ANOVA) to investigate differences between countries with regard to organization of homeschooling. Effects sizes were calculated using eta squared (*η*^2^), where 0.14 is considered a large effect, 0.06 a medium effect, and 0.01 a small effect [12]. Second, we used Chi-square analyses to study group differences between the seven countries with regard to organization of support for children with special educational needs (SEN). Effect sizes were calculated using Cramer’s *V*, where 0.50 is considered a large effect, 0.30 a medium effect, and 0.10 a small effect [12]. Third, we used Chi-square analyses, to investigate differences between families with a child with a mental health condition (i.e., MHC group) or without a mental health condition (NO-MHC). For these Chi-square analyses, we first conducted group comparisons for the entire sample, and then separate group comparisons within each country. These Chi-square analyses were also complemented with effect size calculations using Cramer’s *V*. For all analyses, we adjusted the alpha level to *p* < 0.001 to control for multiple comparisons. To reduce the risk of overemphasizing significant effects with small effect sizes, we focused on effects of at least medium size.

## Results

Table 1 presents descriptive data, including the number of families with a child with or without a mental health condition for each country. A significant medium-sized effect of child age was found. The children of the Swedish participants were older than the children in the other countries, because in Sweden homeschooling was mainly implemented for children ≥ 15 years. There were also large differences between some countries with regard to the proportion of families with a child with mental health problems. This was a result of the fact that families with mental health problems were over-sampled in all of the included countries, except Germany and Italy. As mental health problems are likely to affect the experiences of homeschooling, we therefore only included families without mental health problems when examining differences between countries.

**Table 1:** Descriptive statistics for background variables and results of Chi-square analyses and effects sizes (ES) for differences between countries

	Total	UK	Sweden	Spain	Belgium	Netherlands	Germany	Italy	*χ*^2^ (ES)
Number of participants, *n*	6720	509	1432	1491	508	324	1662	794	
Child age									2260.94* (0.34)
5–8 years, %	21.1	29.4	3.7	29.3	27.1	24.5	19.6	29.2	
9–12 years, %	34.7	37.4	10.8	41.1	36.1	48.6	41.0	43.5	
13–16 years, %	28.3	31.8	31.4	23.5	28.0	23.2	33.4	20.9	
17–19 years, %	16.0	1.4	54.1	6.0	8.8	3.8	6.0	6.5	
Child sex, % females	46.8	38.9	47.8	47.5	47.6	46.4	48.0	45.5	14.80 (0.05)
Rater sex, % females	88.5	94.1	88.3	87.9	87.8	87.8	85.8	93.1	45.30* (0.08)
Immigrant background, %	4.3	1.2	4.4	7.1	5.5	2.3	3.6	2.2	55.00* (0.09)
Parental education, %									
Secondary school or less		5.7	0.8	12.1	0.4	0.0	1.8	6.1	
High school or equivalent		19.5	16.6	23.4	13.8	29.6	19.7	34.7	
University		74.8	82.6	64.5	85.8	68.8	78.3	59.3	
Parental occupation, %									1132.52* (0.24)
Working full-time	48.5	36.9	69.3	50.0	55.5	27.5	33.8	50.8	
Working part-time	29.8	41.8	13.6	13.7	33.3	53.1	49.9	27.4	
Unemployed (looking for employment)	4.1	1.9	1.5	10.9	1.4	3.7	1.8	4.7	
Other (e.g., housewife, retired)	17.5	19.7	15.5	25.4	9.8	15.7	14.5	17.1	
Child mental health condition, %^b^	29.4	37.4	45.6	31.4	35.4	48.8	10.8	18.6	587.15* (0.30)
Attention deficit hyperactivity disorder	12.3	17.9	25.7	7.9	14.4	16.7	5.3	4.3	
Autism spectrum disorder	6.8	9.8	17.3	1.5	8.1	18.2	1.7	0.8	
Dyslexia	9.8	10.4	10.4	18.3	13.2	16.4	0.4	7.3	
Depression/anxiety	5.2	8.3	12.9	2.7	2.6	3.7	2.5	2.0	
Other (e.g., social phobia, language disorder, anorexia, obsessive compulsive disorder)	8.3	11.2	10.4	8.2	12.8	19.4	3.1	6.7	

*Significant at *p* < 0.001

^a^The percentages add up to more than 100%, as a relatively large proportion of children with mental health conditions had several diagnoses

### Differences between countries

#### Organization of homeschooling

On average, children spent about 50% of their school time on self-studies and about 30% in contact with a parent (Table 2). Thus, the time spent in contact with a teacher or with peers was very limited. When comparing the percentage of time in contact with teachers, a number of medium-to-large effect sizes were found. Percentages were the lowest in UK and Germany (about 5% each), and these percentages differed from Spain, Belgium and the Netherlands (11.83–13.93%), which in turn differed from Italy and Sweden (24.38% and 30.12%, respectively). For peer contact, students in Sweden (12.87%) and Belgium (12.43%) spent more time studying with peers compared to students in the UK (3.06%). Students in Italy (35.16%) spent less time with self-study compared to students in Belgium (54.07%) and Germany (54.53%). Finally, students in Sweden (15.28%) spent less time studying with a parent compared to students in all other countries (30.19–45.18%).

**Table 2:** Results for organization of homeschooling as well as results of ANOVAs and effects sizes (ES) for differences between countries

	Total	UK	Sweden	Spain	Belgium	Netherlands	Germany	Italy	*F* (ES)
Percentage of time spent (SD) on different activities during homeschooling^a^									
Contact with teacher (e.g., live webinars)	13.71 (17.64)	4.41^c^ (9.54)	30.12^a^ (22.27)	12.58^b^ (15.49)	11.83^b^ (11.47)	13.93^b^ (14.09)	5.16^c^ (8.49)	24.38^a^ (19.68)	326.62* (0.29)
Contact with peers (e.g., working in small groups)	7.68 (11.25)	3.06^b^ (7.44)	12.87^a^ (11.70)	11.70 (12.43)	12.43^a^ (8.73)	6.96 (10.58)	6.28 (10.82)	7.36 (19.67)	48.00 (0.06)
Self-study (e.g., individual assignments)	47.52 (26.95)	48.44 (34.36)	43.26 (22.83)	43.62 (28.26)	54.07^a^ (25.62)	47.27 (25.45)	54.53^a^ (25.54)	35.16^b^ (23.01)	51.41 (0.06)
Contact with parent (e.g., assisting in schoolwork)	29.85 (29.58)	45.18^a^ (35.21)	15.28^b^ (22.99)	37.25^a^ (31.20)	30.19^a^ (27.46)	33.33^a^ (27.81)	33.99^a^ (27.32)	35.24^a^ (29.85)	66.91 (0.08)
Special educational needs (SEN)									*χ*^2^
Percentage of children with SEN									
MHC group	63.0	60.3	76.3	58.4	58.9	56.7	35.6	66.4	117.26* (0.24)
NO-MHC group	7.4	3.8	7.3	11.9	6.1	18.1	6.3	2.8	86.86* (0.14)
Children with SEN who also receive special education support during homeschooling	78.4	79.4	79.9	79.4	80.2	79.8	71.3	73.2	7.86 (0.07)
Children receiving extra support during homeschooling whose parents feel that this support is not sufficient	65.3	72.5	62.1	66.4	74.6	69.2	61.3	49.1	15.97 (0.12)
Children with SEN, where the school has not been in contact with the family to discuss homeschooling	45.5	33.6	55.7	29.0	48.8	35.0	60.1	51.8	92.15* (0.24)

The letters a and b indicate a significant difference between countries with an effect size of at least medium size (*V* ≥ 0.30), with the letter a always indicating a larger proportion compared to countries marked with the letter b, and c the lowest proportion

*Significant at *p* < 0.001

^a^With regard to homeschooling organization, we had some technical problems with the survey, resulting in loss of data in all countries except Germany. For these analyses, the number of participants for these analyses is therefore lower. UK: *n* = 509; Sweden: *n* = 1432; Spain: *n* = 1491; Belgium = 508; Netherlands: *n* = 324; Germany: *n* = 1662; Italy: *n* = 794. However, the sample with missing data for this question did not differ significantly from the remaining sample with regard to any of the background variables

Except for self-study, significant medium-to-large-sized effects of age group were found, *F* > 225.58, *p* < 0.001. Time spent in contact with teachers and peers increased with age, whereas the opposite pattern was found for time spent with parents. However, even among teenagers, the average percentage for teacher contact was as low as 4.42% in the UK and 6.90% in Germany, but 34.16% in Sweden and 36.22% in Italy.

With regard to SEN, 7.4% of the parents in the NO-MHC group and 60.3% of the parents in the MHC group reported that their child had SEN. Most children with such needs (78.4%) received extra educational support during homeschooling, with similar percentages across countries (Table 2). However, a majority (65.3%) did not feel this support was sufficient, and many schools (29.0–55.7%) had not been in contact with parents to discuss the need for extra educational support during homeschooling. No significant differences between countries of at least medium effect size were found for special education.

#### Positive and negative experiences of homeschooling

A significant effect of country was found for all variables related to positive and negative experiences of homeschooling (see Table 3, NO-MHC group). The proportion of parents reporting “the quality of my child’s homeschooling is very poor” was, on average, 19.2%. Significant group differences with a medium effect size were found on this variable between Belgium and Sweden (both about 8%) and Spain (31.3%). A relatively large proportion of parents (18–45%) reported that schools’ support to students during homeschooling had been insufficient, with no medium-sized effects when comparing different countries. On average, 16.7% of the parents reported that “it is impossible to get homeschooling to work well.” This proportion differed between countries with lower percentages in Belgium and Sweden (< 5%) compared to Spain (23.7%) and Italy (26.6%).

**Table 3:** Effects of homeschooling in families with a child with a mental health condition (MHC) and without a mental health condition (NO-MHC), results of Chi-square and effect size for the differences between countries and effect sizes for the comparison between the MHC group and the NO-MHC group

		TOTAL	UK	Sweden	Spain	Belgium	Netherlands	Germany	Italy	Country differences *χ*^2^ (ES)	NO-MHC vs MHC group *χ*^2^ (ES)
Quality of homeschooling											
1. Quality of homeschooling very poor	MHC	24.1	30.5	14.7	37.6	17.2	17.2	26.1	28.1	214.11* (0.21)	20.21* (0.06)
	NO-MHC	19.2	14.6	8.1^b^	31.3^a^	8.2^b^	12.1	17.9	26.1		
2. School’s support is not sufficient	MHC	45.1	36.0	39.7	55.6	40.6	45.6	51.7	44.5	225.70* (0.22)	40.71* (0.08)
	NO-MHC	36.8	22.0	18.2	42.2	28.7	31.5	45.2	43.7		
3. Impossible to get homeschooling to work well	MHC	26.9	38.8	18.8	36.9	7.2	29.1	25.6	38.8	210.63* (0.21)	90.78* (0.12)
	MHC	16.7	17.9	4.4^b^	23.7^a^	3.0^b^	19.4	16.7	26.6^a^		
General experiences											
4. Negative effects on the child’s life	MHC	33.1	33.3	34.0	32.4	31.7	23.4	37.2	38.5	33.50 (0.08)	63.29* (0.10)
	NO-MHC	24.6	22.2	17.4	23.4	24.7	18.1	27.6	24.6		
5. Negative effects on the parent’s life	MHC	56.2	56.6	32.8	48.8	48.3	33.5	53.9	52.7	215.45* (0.21)	71.04* (0.10)
	NO-MHC	32.9	36.8^a^	11.1^b^	38.5^a^	33.5^a^	27.7	36.6^a^	41.3^a^		
6. Positive effects on the child’s life	MHC	34.9	39.4	38.9	31.5	31.1	51.0	26.1	19.9	126.92* (0.16)	91.94* (0.12)
	NO-MHC	23.4	27.2	32.6	25.8	23.8	38.0^a^	19.8	11.1^b^		
7. Positive effects on the parent’s life	MHC	23.9	25.9	25.6	23.5	22.8	36.3	16.1	12.1	163.08* (0.19)	15.37* (0.05)
	NO-MHC	19.6	31.3	25.0	21.7	19.2	38.6^a^	16.5	6.3^b^		
Parental worry/stress											
8. Parental worry about child performing worse	MHC	51.6	60.5	42.9	64.5	52.2	30.6	47.2	64.9	317.49* (0.26)	112.28* (0.13)
	NO-MHC	37.6	29.8	18.1^b^	55.3^a^	40.5	29.7	33.1	48.0^a^		
9. Parental stress due to extra workload	MHC	57.6	69.5	40.1	71.2	67.8	50.0	62.2	66.2	471.13* (0.32)	36.33* (0.07)
	NO-MHC	49.5	58.5	14.2^b^	56.8^a^	57.6	47.6	57.2	54.9^a^		
Childs ability to participate in homeschooling											
10. Too high demands on planning own schoolwork	MHC	53.0	54.0	50.7	59.5	51.1	47.5	54.4	48.3	98.22* (0.14)	216.39*(0.18)
	NO-MHC	33.8	24.2	21.5	38.6	35.4	33.7	39.4	32.1		
11. Cannot fully participate in homeschooling	MHC	27.7	44.4	23.3	29.9	48.9	12.7	18.9	18.9	219.10* (0.22)	264.80* (0.20)
	NO-MHC	11.6	19.3	5.3^b^	15.5	31.4^a^	5.4^b^	8.0^b^	8.8^b^		

Numbers indicate percentage of parents reporting a score of 4 or 5 (“more” or “much more”). The letters a and b indicate a significant difference between countries with an effect size of at least medium size (*V* ≥ 0.30), with the letter a always indicating a larger proportion compared to countries marked with the letter b

*Significant at *p* < .001

**Fig. 2:**
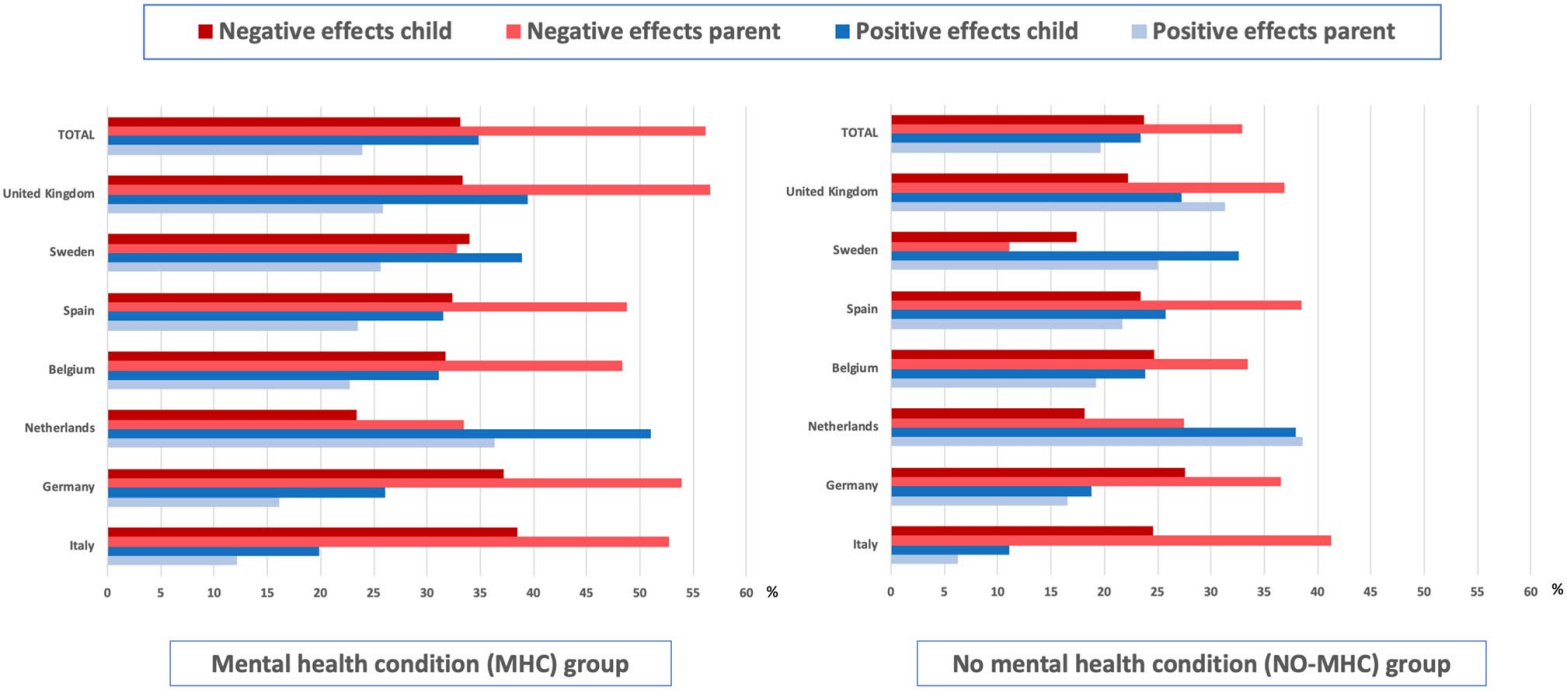
Percentage of parents reporting general negative and positive effects of homeschooling on their child and themselves, presented separately for the MHC group and the NO-MHC group

Parents frequently reported general negative experiences of homeschooling for both their child (17.4–27.6%) and themselves (11.1–41.3%). In all countries except Sweden, parents reported higher levels of positive experiences for themselves compared to their child (see Fig. 2). Differences between countries were generally small, except that the proportion of parents reporting negative experiences of homeschooling for parents were lower in Sweden (11.1%) compared to all other countries except the Netherlands (33.5–41.3%).

A substantial proportion of parents also reported positive experiences of homeschooling for children (11.1–38.0%) and themselves (6.3–31.1%). As can be seen in Fig. 2, the proportion of parents in the NO-MHC group reporting positive experiences of homeschooling were actually somewhat higher compared to those reporting negative experiences in both Sweden and the Netherlands, but not in the other five countries. With regard to positive experiences for children, differences between countries with medium-sized effects were found between Italy (11.1%) and the Netherlands (38.0%). With regard to positive experiences of homeschooling for parents themselves, there was a difference between Italy (6.3%) and the Netherlands (38.6%).

For parental worry and stress, the proportions exceeded 40% in many countries. Medium-size effects were found for both parental worry and stress when comparing Sweden (18.1% and 14.2%) with Spain and Italy (48.0–56.8%).

Across countries, about one third (33.8%) of parents felt homeschooling put too high demands on children. Some parents (11.6%) also reported that their child was unable to fully participate in homeschooling. More parents in Belgium (31.4%) than in Sweden, the Netherlands, Germany, and Italy (5.3–8.8%) reported that their child could not fully participate in homeschooling (Fig. 2).

No significant effects of age were found for negative or positive effects on children or positive effects on parents. For all other homeschooling-related variables, parents of younger children reported significantly more problems compared to parents of older children, all *F* > 20.07, *p* < 0.001. However, effect sizes were small (all *η* < 0.06), except for a large effect of age for parental stress.

#### Changes in daily life functioning during the COVID-19 pandemic

Questions related to daily functioning asked participants to compare their current situation with the situation before the COVID-19 pandemic. Thus, these questions were not meant to capture experiences of homeschooling specifically, but rather changes during the COVID-19 pandemic in general. In most countries, a large proportion of parents (most often 50% or above) reported that they and their child felt more isolated and that the child used digital media more often for things besides schoolwork (Table 4, NO-MHC group). Differences between countries were significant, although effect sizes were generally small, except for medium-size effects showing that fewer children in Sweden (35.9%) increased their digital media use compared to children in the UK, Spain and Belgium (68.7–75.8%). Concerning potentially more serious negative aspects, a relatively large proportion of parents reported more conflicts with the child (11.0–41.3%) and between adults (6.8–22.5%). A smaller proportion of parents (on average 5%) also reported increased levels of parental alcohol/drug use, with medium-sized effects when comparing the UK (19.1%) with Sweden, Spain and Italy (> 3%). Finally, a substantial proportion of parents reported changes in work-related problems (10.9–29.9%) or financial problems (7.4–20.0%) during the COVID-19 pandemic. None of the differences between countries reached a medium effect size.

No significant effects of child age were found for parental alcohol/drug use, increased conflicts between adults or financial problems. For all other variables related to effects on daily life functioning, parents of younger children reported significantly more problems compared to parents of older children, all *F* > 5.24, *p* < 0.001, though effect sizes were small (*η* < 0.02).

**Table 4:** Experiences on daily life functioning in families with a child with a mental health condition (MHC) and without a mental health condition (NO-MHC), results of Chi-square and effect size for the differences between countries and effect sizes for the comparison between the MHC group and the NO-MHC group

		Total	UK	Sweden	Spain	Belgium	Netherlands	Germany	Italy	Country differences *χ*^2^ (ES)	NO-MHC vs MHC group *χ*^2^ (ES)
1. Child feels socially isolated	MHC	55.4	66.0	53.5	44.1	69.7	52.2	66.3	57.6	156.32* (0.18)	14.52* (0.05)
	NO-MHC	60.4	67.3	55.7	47.8	76.9	48.2	67.3	61.3		
2. Parent feels socially isolated	MHC	57.0	69.2	56.0	46.3	69.3	63.7	61.2	50.7	103.15* (0.15)	0.06 (0.00)
	NO-MHC	57.2	67.2	53.2	47.9	72.7	54.9	62.0	53.4		
3. Adults drink alcohol/use other drugs	MHC	5.0	16.0	1.3	2.9^b^	14.1	3.9	6.1	1.7^b^	176.62* (0.20)	0.01 (0.00)
	NO-MHC	5.0	19.1^a^	2.1^b^	2.5^b^	9.1	5.0	5.7	1.5^b^		
4. Conflicts with the child	MHC	30.7	40.4	23.8	30.8	35.2	30.4	45.8	24.1	266.51* (0.24)	2.08 (0.02)
	NO-MHC	28.9	38.4	11.0	27.4	28.1	25.0	41.3	20.8		
5. Conflicts between adults	MHC	14.6	18.8	10.3	15.9	14.9	9.0	28.7	12.3	132.65* (0.17)	0.04 (0.00)
	NO-MHC	14.8	18.7	6.8	12.8	13.2	6.2	22.5	10.4		
6. Digital media use, not including schoolwork	MHC	60.9	74.5	48.8	66.7	75.8	60.1	57.1	65.8	286.72* (0.25)	0.31 (0.00)
	NO-MHC	60.2	75.0^a^	35.9^b^	68.7^a^	73.7^a^	51.2	59.8	64.7		
7. Work-related problems	MHC	24.1	33.0	13.4	34.4	15.3	21.4	32.6	33.1	102.13* (0.15)	2.32 (0.02)
	NO-MHC	22.3	28.1	10.9	27.5	17.4	19.9	22.0	29.9		
8. Financial problems	MHC	17.8	16.5	13.1	24.0	10.2	13.5	26.5	26.2	82.74* (0.14)	7.58 (0.04)
	NO-MHC	15.0	14.3	7.4	20.0	8.4	7.4	16.5	19.5		

Numbers indicate percentage of parents reporting a score of 4 or 5 (“more” or “much more”). The letters a and b indicate a significant difference between countries with an effect size of at least medium size (*V* ≥ .30), with the letter a always indicating a larger proportion compared to countries marked with the letter b

*Significant at *p* < .001

### Differences between families with or without a child with a mental health condition

As seen in Table 3, differences between the MHC and the NO-MHC group were significant for all variables related to parental experience of homeschooling, with the MHC group reporting more negative experiences, but also more positive experiences, compared to the NO-MHC-group. However, the effect sizes for these differences were mostly small. Medium effect sizes were only found between the MHC and the NO-MHC group in the UK and Sweden for the question on parental worry and the two variables related to child participation in homeschooling. In addition, there were differences of medium effect sizes in Sweden for parental stress and negative effects of homeschooling for the parent. Regarding effects on daily life functioning (see Table 4), the proportion of parents reporting negative experiences were very similar in the MHC and NO-MHC group. The only significant difference was that more parents in the MHC reported that their child felt socially isolated compared to parents in the NO-MHC group. However, the effect size was small and no medium-sized effects were found when comparing the MHC group and the NO-MHC groups separately for each country.

## Discussion

This is, to our knowledge, the first large-scale European study assessing parental experiences of homeschooling on families during the COVID-19 pandemic, as well as identifying areas of greater impact for families with a child with a mental health condition. Results indicate that parents across Europe reported negative experiences for both themselves and their children. This included increases in domestic conflict, parental alcohol/drug use and poor-quality homeschooling. A proportion of parents in each country reported that their child was unable to participate in homeschooling. It is likely that these children may fall behind academically without appropriate support. The majority of parents of children with SEN reported receiving no or insufficient support during homeschooling. Parents of children with mental health conditions in our study reported significantly more negative experiences of homeschooling, but not on overall daily life functioning. However, most effect sizes were small, with parents of a child with a mental health condition primarily reporting more worry, stress and greater difficulties with child participation in homeschooling in some countries. Some parents also had positive experiences of homeschooling for both themselves and their child.

With regard to organization of homeschooling, results indicated that schools in most countries did not adjust to online teaching during school closures during the first wave of the COVID-19 pandemic. In Sweden and Italy, digital platforms and online teaching were available for teenagers. However, in the UK and Germany, parents reported that children of all ages had no or very limited contact with teachers during homeschooling, and online teaching was reported to be very limited in the other countries as well. Thus, parents have been primarily responsible for schooling in many European countries during school closures, which may ultimately lead to increased disparities in educational progress. In addition, children of low socioeconomic status may be exposed to other negative effects not investigated in the present study. Previous research has for example shown that, even during summer vacation, children’s well-being can be compromised regarding access to healthy food, personal safety, and emotional support [13]. Thus, we may have to prepare for additional long-term negative effects related to the COVID-19 pandemic’s general impact on society as a whole. Increased differences in students’ knowledge will pose challenges for teachers regarding meeting the needs of individual children when schools re-open.

A relatively large proportion of parents also reported that homeschooling was of low quality, with low levels of support from schools and general negative effects of homeschooling on both children and parents. Parents also reported increases in parental stress/worry and domestic conflict during the COVID-19 pandemic. Our results are in line with previous commentaries [2, 3] as well as some smaller empirical studies [4, 5] emphasizing that the COVID-19 pandemic is likely to have major effects on daily life functioning for both children and adults.

With mostly small, although significant, effects between countries, our findings indicate that many parents in all seven countries reported negative experiences of homeschooling. With regard to the few medium-size differences found between countries, more negative parental experiences were generally found for families with younger compared to older children, which is in line with a previous study [14]. This could explain why Swedish parents, who primarily had children ≥ 16 years at home, reported lower levels of general negative effects on parents, as well as lower levels of parental worry and stress, compared to parents in several other countries. Sweden has also not experienced the same shutdown of society during the COVID-19 pandemic that the other countries included in the study have. A German study [15] showed that changes in school regulations, exams and school activities due to the pandemic have been so heterogenous, both between and within countries, that a general overview of measures taken by the countries included in our study is not possible. Thus, it was unfortunately not possible to conduct more detailed analyses linking our findings to the degree of lockdown in different countries.

Regarding families with a child with mental health problems**,** previous commentaries [6, 7] as well as other empirical studies [8–10] have suggested that homeschooling is more challenging for them. This may be because homeschooling increases demands on executive functioning, which involves skills associated with mental health problems, especially neurodevelopmental disorders [16]. Surprisingly, our results showed mostly small effect sizes when comparing the MHC and the NO-MHC group. Thus, adverse effects were generally reported in families both with and without children with mental health problems. However, a larger proportion of children with a mental health condition was unable to fully participate in homeschooling, implying that children in the MHC group received less schooling than the other students during the many weeks of homeschooling. In addition, 63% of the children with a mental health condition had SEN, and the majority of parents reported that extra support during homeschooling had either been non-existent or insufficient. These children are therefore at risk of falling further behind their peers academically, and previous studies have also shown that academic failure mediates the relation between ADHD and depression [17].

Furthermore, serious concerns have been raised that the COVID-19 pandemic will lead to inadequate treatment for children with mental health problems (e.g., canceled treatments, delays in titration and optimization of medication) [18, 19]. Thus, the negative effects of homeschooling on children with mental health problems found in the present study further exacerbate the increased burden these families are experiencing during the COVID-19 pandemic.

With regard to potential positive effects of homeschooling, previous research [3, 12] has suggested that children troubled by school due to bullying or other stressors may experience homeschooling as a relief. Interestingly, our results showed that while parents of children with mental health conditions reported overall more negative effects of homeschooling, they also reported more positive effects. We believe that this may be a result of the fact that for some children with mental health problems, homeschooling result in fewer disturbances, more flexibility in organizing schoolwork, and less anxiety due to decreased contact with peers and in some countries decreased exam pressure.

### Strengths and limitations

The present study has the advantage of including a large sample from seven European countries. Moreover, the assessments were made during school shutdowns rather than relying on retrospective reports. Furthermore, oversampling parents of children with mental health problems allowed us to examine the effect of COVID-19 on vulnerable groups, as emphasized in prior research [14, 20].

One limitation of the present study was that we relied on parents’ judgements of their current daily life functioning compared to before the pandemic rather than collecting longitudinal data. Thus, we did not know to what extent parents had negative experiences of their child’s education also before the pandemic. However, previous research [15] has clearly showed that the quality of homeschooling during the first wave of the COVID-19 pandemic was perceived to be lower than regular schooling. Another aspect that could be seen as a limitation is that we did not use standardized, validated measures. However, we designed the survey based on a previous qualitative COVID-19 study we conducted with parents where they described the effects of homeschooling on family functioning. This study revealed a number of themes and there was no questionnaire available that captured all relevant aspects. In addition, including a large number of different scales would have increased the survey length substantially and this would most likely have decreased the response rate, especially among families with mental health problems.

Because we used anonymous surveys distributed through social media, we lack some important information on the characteristics and selection of the sampled population. However, families with a low level of parental education and immigrant background were clearly underrepresented. This was despite attempts to recruit via schools in a range of socio-economic areas in each country, suggesting that parents from lower socio-economic groups chose not to participate in the study. Also, the participants with immigrant background were mainly from other European countries. As it has been argued that families with low socioeconomic backgrounds are especially vulnerable to the negative consequences of COVID-19 [14, 21], the present study may have underestimated the negative effects of homeschooling, especially in specific vulnerable groups. Furthermore, the results of the study mainly apply to non-immigrant families with parents of medium to high levels of education and are therefore limited in their generalizability to other sectors of the population in each country. Relying on parental reports, we are unable to validate whether children met the full diagnostic criteria of the mental disorder the parents reported. Furthermore, in this short survey, we were unable to assess a broader range of factors contributing to negative outcomes of homeschooling (e.g., parental psychopathology, long-term effects of homeschooling) and information from several sources (e.g., adolescents and teachers).

## Conclusions

Using school closures as a means to reduce the spread of COVID-19 has been questioned, as recent reports have suggested that children are unlikely to be the main drivers of the pandemic [22]. Thus, compared to other forms of social distancing, school closures likely have more limited effects on transmission of COVID-19 [23]. The present results clearly show that parents experienced that homeschooling had adverse effects on both parents and children, although some positive experiences were also reported. The potential positive effects of homeschooling need to be further explored. As emphasized by previous research [24, 25], homeschooling during the COVID-19 pandemic has given schools an opportunity to rethink education and consider not only challenges but also opportunities related to the use of digital teaching. However, it is also important to consider that online teaching can lead to increased inequalities between children, further exposing those with a low socioeconomic background to adverse effects. A recent OECD-report [26] showed that schools have faced many challenges during COVID-19 school closures (e.g., poor availability of effective online platforms and poor technical skills among teachers). The report also emphasized that closing schools “has shed light on inequalities related to access to education, and on student well-being in the absence of social interactions and social services provided in schools” (p. 32). It is therefore crucial that policymakers balance potential benefits and negative effects before imposing new school lockdowns during later phases of the COVID-19 pandemic or future pandemics.
